# Supplementary material for: Genes Involved in DNA Repair and Mitophagy Protect Embryoid Bodies from the Toxic Effect of Methylmercury Chloride under Physioxia Conditions
Source: Cells. 2023 Jan 21;12(3):390. doi: 10.3390/cells12030390 (PMC9913246; doi:10.3390/cells12030390)
Supplement: Supplementary file 1 [file cells-12-00390-s001.zip › Table S1 Summary of the influence of MeHgCl on hiPSCs under 21% O2.pdf]

Table S1: Summary of the influence of MeHgCl on hiPSCs under 21% O_2 conditions („+" presence of correctly formed EBs was confirmed, „-" absence of correctly formed EBs was confirmed) (*, $p<0.5$; **, $p<0.01$; „ns"-statistically insignificant)

Tested parameter	0 µM	0.125 µM	0.25 µM	0.5 µM	1 µM
Apoptosis	ns	ns	ns	ns	↑(**)
Viablility	ns	ns	ns	ns	ns
ROS level	ns	ns	ns	ns	↑ (*)
Mitochondrial membrane potential	ns	ns	ns	ns	↓(*)
Ability for EBs formation (24h of exposure)	+	+	+	+	-
Ability for EBs differentiation (7 days of exposure)	+	-	-	-	-
