# Supplementary material for: Genes Involved in DNA Repair and Mitophagy Protect Embryoid Bodies from the Toxic Effect of Methylmercury Chloride under Physioxia Conditions
Source: Cells. 2023 Jan 21;12(3):390. doi: 10.3390/cells12030390 (PMC9913246; doi:10.3390/cells12030390)
Supplement: Supplementary file 1 [file cells-12-00390-s001.zip › Table S2 Summary of the influence of MeHgCl on EBs under 21% O2 conditions.pdf]

Table S2: Summary of the influence of MeHgCl on EBs under 21% O_2 conditions („+” presence of correctly formed EBs was confirmed, „-„ absence of correctly formed EBs was confirmed)

Exposure time	0 µM	0.125 µM	0.25 µM	0.5 µM
24h	+	+	+	+
4 days	+	+	+	-
7 days	+	+	-	-
14 days	+	-	-	-
