# Supplementary material for: Genes Involved in DNA Repair and Mitophagy Protect Embryoid Bodies from the Toxic Effect of Methylmercury Chloride under Physioxia Conditions
Source: Cells. 2023 Jan 21;12(3):390. doi: 10.3390/cells12030390 (PMC9913246; doi:10.3390/cells12030390)
Supplement: Supplementary file 1 [file cells-12-00390-s001.zip › Table S3 Summary of the mtDNA copy number and AP sites number in EBs after MeHgCl treatment under 21%O2 or 5%O2 conditions.pdf]

Table S3: Summary of the mtDNA copy number and AP sites number in EBs after MeHgCl treatment under 21%O_2 or 5%O_2 conditions (*, $p<0.5$; **, $p<0.01$; ***$p<0.001$; „ns”- statistically insignificant)

Tested parameter	*5%O_2 vs. 21%O_2*	*21%O_2+MeHgCl vs. 21%O_2*	*5%O_2+MeHgCl vs. 5%O_2*	*5%O_2+MeHgCl vs. 21%O_2+MeHgCl*
mtDNA copy number	↓ (***)	↓ (*)	ns	↓ (***)
AP sites	ns	↑ (*)	ns	ns
