# Supplementary material for: Genes Involved in DNA Repair and Mitophagy Protect Embryoid Bodies from the Toxic Effect of Methylmercury Chloride under Physioxia Conditions
Source: Cells. 2023 Jan 21;12(3):390. doi: 10.3390/cells12030390 (PMC9913246; doi:10.3390/cells12030390)
Supplement: Supplementary file 1 [file cells-12-00390-s001.zip › Table S4 Summary of the gene expression changes in EBs cultured under 21% O2 or 5% O2 condi-tions.pdf]

Table S4: Summary of the gene expression changes in EBs cultured under 21% O_2 or 5% O_2 conditions (*,p<0.5; **, p<0.01; *** p<0.001, ns-statistically insignificant)

Gene	*5%O_2 vs. 21%O_2*
NES	↓(***)
SOX17	↓(***)
TBXT	ns
TUBB3	↑(***)
TFAM	↓(**)
POLG1	↑(**)
PARK2	↑(***)
ATM	↑(***)
PARP1	↑(***)
OGG1	↑ (*)
