# Supplementary material for: Genes Involved in DNA Repair and Mitophagy Protect Embryoid Bodies from the Toxic Effect of Methylmercury Chloride under Physioxia Conditions
Source: Cells. 2023 Jan 21;12(3):390. doi: 10.3390/cells12030390 (PMC9913246; doi:10.3390/cells12030390)
Supplement: Supplementary file 1 [file cells-12-00390-s001.zip › Table S5 Summary of the gene expression changes in EBs after MeHgCl treatment under 21% O2 or 5% O2 conditions.pdf]

Table S5: Summary of the gene expression changes in EBs after MeHgCl treatment under 21% O_2 or 5% O_2 conditions (*,p<0.5; **, p<0.01; *** p<0.001; ns-statistically insignificant)

Gene	*21%O_2+MeHgCl vs. 21%O_2*	*5%O_2+MeHgCl vs.5%O_2*	*5%O_2+MeHgCl vs. 21%O_2+MeHgCl*
NES	ns	ns	ns
SOX17	↓ (*)	ns	ns
TBXT	↑ (*)	ns	ns
TUBB3	↑ (*)	ns	↓ (*)
TFAM	ns	↑ (*)	ns
POLG1	↓ (*)	↓ (*)	ns
PARK2	↓ (*)	ns	↑ (***)
ATM	ns	ns	ns
PARP1	ns	↓ (**)	ns
OGG1	ns	↓ (**)	ns
