# Supplementary material for: Genes Involved in DNA Repair and Mitophagy Protect Embryoid Bodies from the Toxic Effect of Methylmercury Chloride under Physioxia Conditions
Source: Cells. 2023 Jan 21;12(3):390. doi: 10.3390/cells12030390 (PMC9913246; doi:10.3390/cells12030390)
Supplement: Supplementary file 1 [file cells-12-00390-s001.zip › Table S6 Summary of the changes in selected developmental markers in EBs after MeHgCl treatment under 21% O2 or 5% O2 conditions.pdf]

Table S6: Summary of the changes in selected developmental markers in EBs after MeHgCl treatment under 21% O_2 or 5% O_2 conditions („-„ no expression, „+”low expression, „++” medium expression, „+++” high expression) as revealed by qualitative immunocytochemistry study.

Markers	*21%O_2*	*5%O_2*	*21%O_2+MeHgCl*	*5%O_2+MeHgCl*
Oct4	-	-	-	-
Nanog	-	+	+	-
Pax6	++	+	+	-
Nestin	+++	++	+	-
NF-200	+++	++	*presence of single cells expressing NF-200*	*presence of single cells expressing NF-200*
FOXA2	+	++	+	+
αSMA	+++	+	*presence of single cells expressing αSMA*	-
γH2AX	*presence of single γH2AX foci*	*presence of single γH2AX foci*	*presence of single γH2AX foci*	*presence of single γH2AX foci*
Casp3	*presence of single cells expressed Casp3*	*presence of single cells expressed Casp3*	+++	++
Ki67	*presence of single cells expressed Ki67*	*presence of single cells expressed Ki67*	*presence of single cells expressing Ki67*	*presence of single cells expressing Ki67*
Nucleus morphology	*lack of changes*	*lack of changes*	*chromatin condensation, nucleus shrinkage, DNA fragmentation*	*lack of changes*
